# Safety and Efficacy of Insulin and Heparin in the Management of Hypertriglyceridemia-Induced Pancreatitis in a Patient without Diabetes: A Case Report

**DOI:** 10.1155/2022/7905552

**Published:** 2022-09-27

**Authors:** Luis Tolento Cortes, Jessica Trinh, Mimi Le, Philip Papayanis, Leah Tudtud-Hans, Lisa Hong

**Affiliations:** ^1^Loma Linda University Health, School of Pharmacy, 24745 Stewart St, Loma Linda, CA, USA; ^2^Loma Linda University Health, School of Medicine, 11175 Campus St, Loma Linda, CA, USA

## Abstract

Acute pancreatitis (AP) leads to a variety of complications, such as local or systemic inflammatory responses as well as organ failure. While choledocholithiasis and alcohol abuse are two of the most common causes of AP, hypertriglyceridemia causes AP with an incidence rate between 2 and 5%. The management of hypertriglyceridemia-induced pancreatitis (HTGIP) is focused on the lowering of triglyceride (TG) levels, and the efficacy of therapies for the management of HTGIP may vary based on the hypertriglyceridemia etiology. The aim of this article is to report a case of a 43-year-old female with a history of familial hypertriglyceridemia and without diabetes who was admitted for acute pancreatitis with a TG level elevated to 4,435 mg/dL. The patient was treated with a combination of insulin, heparin, atorvastatin, and omega-3-acid ethyl esters, and her TG level was reduced to 880 mg/dL after 9 days of therapy. Despite the successful treatment of the patient, standardization of the approach for the treatment of HTGIP is needed. Future research should aim to identify the appropriateness of insulin therapy specifically in patients without diabetes presenting with hypertriglyceridemia and the dosing associated with optimal safety.

## 1. Introduction

Pancreatitis is one of the most common gastrointestinal-related disorders, leading to both emergency department visits and hospital readmissions [1]. The prevalence of severe hypertriglyceridemia (triglyceride [TG] levels >500 mg/dL) in adults over the age of 20 years in the United States is approximately 1.7% [2]. Hypertriglyceridemia can have either monogenic or polygenic etiologies, and abnormalities in the LPL, APOC2, APOA5, LMF1, or GPIHBP1 genes can lead to the development of hypertriglyceridemia. Secondary factors include comorbid conditions such as diabetes and obesity. Additionally, lifestyle habits such as alcohol abuse and high-fat diets may also increase the risk of developing hypertriglyceridemia [3, 4]. The mechanism of hypertriglyceridemia-induced pancreatitis (HTGIP) is not well-understood. One theoretical mechanism involves the hydrolysis of triglycerides by pancreatic lipases, which results in the production of free fatty acids (FFA) in the pancreas. This newly hydrolyzed FFA can potentially accumulate in the pancreatic capillary beds. Consequently, the “plugging” of these capillaries may cause ischemia and acidosis. The acidic environment may contribute to the activation of trypsinogen, leading to acinar cell injury and ultimately pancreatitis [5, 6].

Initial management of HTGIP is similar to the management of acute pancreatitis (AP). Therapies tailored to the management of HTGIP involve interventions that can rapidly decrease TG levels. Published reports describe the utilization of agents such as insulin, heparin, fenofibrate, omega-3 fatty acids, and statins, as well as plasmapheresis for TG lowering [7–22]. However, guidance regarding the best practice for pharmacologic treatment in these cases is lacking.

Here we report a case of HTGIP in a patient without diabetes treated with intravenous insulin and subcutaneous heparin. Our case highlights the need for standardization of treatment and close monitoring of adverse events including hypokalemia and hypoglycemia.

## 2. Case Report

A 43-year-old female with a past medical history of familial hypertriglyceridemia, obesity, hyperlipidemia, hypertension, pituitary adenoma, and a recent hysterectomy presented to the emergency department due to worsening left-sided epigastric abdominal pain with associated nausea and decreased appetite. She denied any fever, chills, chest pain, cough, shortness of breath, diarrhea, or urinary symptoms. Family history was positive for diabetes in both parents. The patient denied any history of smoking, alcohol, or illicit drug use.

**Figure 1:**
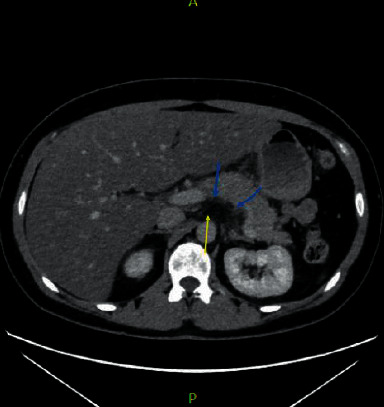
CT abdomen. CT illustrates the blurring of the margins of the pancreas (blue arrows) as well as mild retroperitoneal fat stranding (yellow arrows). These findings are due to inflammation of the fat around the pancreas, which is consistent with the diagnosis of acute interstitial pancreatitis.

Her vital signs demonstrated tachycardia of 119 beats per minute, an elevated blood pressure of 169/111 mmHg, and a respiratory rate of 20 breaths per minute. Physical examination of the abdomen was negative for severe epigastric pain and a left-upper quadrant that was tender to palpation. Laboratory examination revealed serum triglycerides of 4,425 mg/dL, a total cholesterol level of 1,273 mg/dL, high-density lipoprotein <3 mg/dL, lipase of 80 units/L, blood glucose of 256 mg/dL, hemoglobin A1_C_ of 6.0%, potassium of 3.7 mEq/L, as well as aspartate transaminase (AST) and alanine transaminase (ALT) of 73 and 77 units/L, respectively. Given this clinical presentation, there was suspicion of pancreatitis, but the serum lipase was only mildly elevated. Therefore, a computed tomography (CT) scan was performed. The CT showed mild fat stranding around the pancreas, which is consistent with acute interstitial pancreatitis, hepatic steatosis, and nonobstructive cholelithiasis (Figure 1). Investigation with ultrasonography of the right upper quadrant confirmed no gallstone was obstructing the pancreatic duct. Given the patient's severely elevated TGs, no obstruction seen on imaging, and lack of alcohol use disorder, the patient was diagnosed with pancreatitis secondary to hypertriglyceridemia.

Initial treatment included standard fluid resuscitation and pain management with the oral hydrocodone-acetaminophen combination (5 mg and 325 mg) and intravenous morphine (2 mg). The hospital's endocrinology team was consulted for lipid management, and a continuous infusion of regular insulin was initiated at a rate of 10 units/hour. A 2-bag system consisting of 0.9% sodium chloride alone and 5% dextrose/0.9% sodium chloride (both containing potassium phosphate 20 mmol and potassium acetate 20 mEq) was used in conjunction with insulin. The patient was also treated with omega-3-acid ethyl esters at a dose of 2 grams orally twice daily and atorvastatin 20 mg orally daily as formulary substitutions of home medications icosapent ethyl 2 grams orally twice daily and rosuvastatin 5 mg orally daily with a goal of lowering TG to <500 mg/dL. Subcutaneous heparin at 5,000 units every 8 hours was also initiated. The nutrition team was consulted and the patient was maintained on a fat-restricted diet (<30 grams a day). She had 2 days with strict orders to receive nothing by mouth due to severe abdominal pain and her inability to tolerate an oral diet.

**Figure 2:**
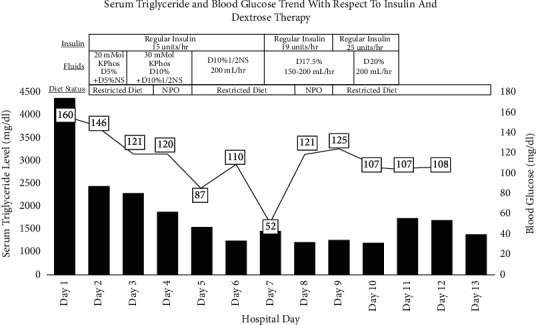
Serum triglycerides and blood glucose during hospital stay.

On hospital day 2, the serum TG dropped to 2,212 mg/dL and the insulin drip was titrated up to 15 units/hour with a dextrose 10% and 0.45% sodium chloride infusion at a rate of 200 mL/hour. The intravenous insulin continued at 15 units/hour until hospital day 7. During this period, the patient's TG levels continued to downtrend, reaching a low of 863 mg/dL (an 80% decrease from baseline) on hospital day 6. On hospital day 7, the patient's TG increased to 1,112 mg/dL (a 74% reduction from baseline). The insulin infusion rate was changed to a weight-based regimen of 0.2 units/kg/hour (patient weight was 97 kg = approximately 19 units/hour) with continuous dextrose 17.5% (started at 150 mL/hour, then titrated to 200 mL/hour) until hospital day 9. While serum TG decreased to 821 mg/dL in response to the increased insulin, three episodes of hypoglycemia were observed within a 9-hour timeframe during the weight-based insulin infusion. An additional 25-gram ampule of dextrose was given to correct these hypoglycemic events. The last insulin infusion titration to 25 units/hour occurred on hospital day 9 with dextrose 20% running at 200 mL/hour and remained at this rate until the termination of insulin therapy on hospital day 10, when the nadir TG level of 809 mg/dL (81.7% reduction from baseline) was obtained. At this time, the endocrinology team recommended discontinuing insulin therapy, and their rationale was that the TG-reducing effect had likely reached its max. The patient remained on heparin for the rest of her hospitalization for continued thromboprophylaxis until hospital day 12. During this period, the TG increased to 1,415 mg/dL on hospital day 11 and decreased to 1,028 mg/dL (76% reduction from baseline) without further therapeutic intervention (see Figure 2).

**Figure 3:**
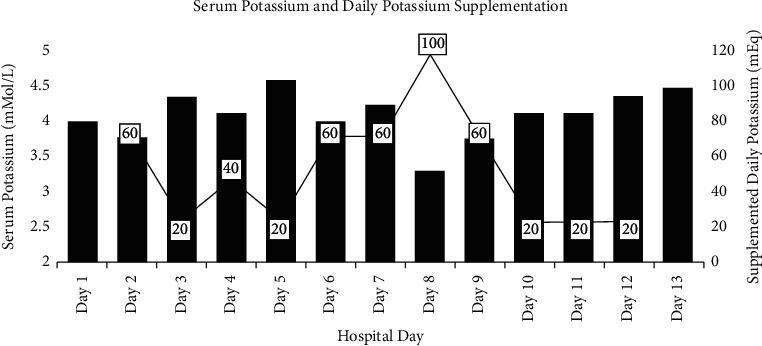
Serum potassium and potassium supplementation.

Due to the effect of insulin therapy on serum potassium levels, potassium was assessed and replenished on hospital days 1 to 4 as appropriate. The patient was started on scheduled potassium replacements of 20 mEq on hospital day 5, but still required several additional doses during the admission (see Figure 3), including 100 mEq of supplemental potassium on hospital day 8 to correct hypokalemia.

The patient was discharged home on hospital day 13 and instructed to continue the triglyceride-lowering oral medications as mentioned above, with the addition of 54 mg fenofibrate orally daily. The patient's TG level was 495 mg/dL during a 1-month postdischarge follow-up appointment.

## 3. Discussion

We report the successful management of a 43-year-old female with a history of familial hypertriglyceridemia without a history of diabetes admitted for acute pancreatitis with the use of continuous insulin infusions and subcutaneous heparin, with serum TG levels falling from 4,425 mg/dL to 809 mg/dL after 8 days of insulin and heparin combination therapy. Triglycerides are normally synthesized in the liver or obtained through exogenous means. Under normal circumstances, these circulating TGs (as chylomicrons and low-density lipoprotein) are metabolized by lipoprotein lipase into FFA. Familial hypertriglyceridemia is polygenic and presents with elevated very low-density lipoprotein (VLDL) levels secondary to impaired catabolism of VLDL. In hypertriglyceridemia, VLDL particles contain a higher percentage of TGs, leading to an increased size of VLDL particles and suboptimal hydrolysis by lipoprotein lipase, which ultimately results in a normal amount of VLDL particles with altered compositions [23].

The role of insulin in the treatment of hypertriglyceridemia is an activation in addition to the synthesis of lipoprotein lipase (LPL). LPL activity is higher in a fed state compared to a fasting state and improves the clearance of TGs from circulation by cleaving TG into FFA, which can be transported into the surrounding tissues for further metabolism or storage. There are also some speculations that insulin may inhibit the re-esterification of FFA into TG [24, 25]. The efficacy of continuous insulin therapy in managing hypertriglyceridemia is referenced in a recent hypertriglyceridemia management article published by the American Family Physician [26]. Success in obtaining significant reductions in TG levels via bolus dosing of insulin has been reported as well [11]. Interestingly, there are no formal recommendations on hypertriglyceridemia management in patients without diabetes.

Heparin has been used in combination with insulin therapy to lower TG levels [27]. The mechanism by which heparin lowers TGs is hypothesized to be due to its ability to release LPL from endothelial cells, preventing LPL from converting to a nonactive unit and decreasing circulating TG. However, there is a concern about rebound hypertriglyceridemia associated with prolonged heparin use due to an imbalance in the production and degradation of LPL [28]. The reported route of heparin administration in the management of hypertriglyceridemia is commonly intravenous, but groups have observed similar efficacy with subcutaneous unfractionated heparin as well as low molecular weight heparin without any reports of bleeding [13, 17].

There is a paucity of data assessing patients without diabetes who present with hypertriglyceridemia. Khan et al. previously reported a successful course of treatment with insulin infusion monotherapy that resulted in a reduction of TG levels from 3,525 mg/dL to 973 mg/dL with only one day of therapy [15]. Gayam et al. also used insulin monotherapy but for a duration of approximately 7 days with a dose of 1–2 units/kg/day (a rate significantly lower than in our case) and observed a reduction in TG levels from >10,000 mg/dL to <300 mg/dL [9]. Another case report by Reed et al. of a patient with familial hypertriglyceridemia and an initial TG level of 3,496 mg/dL demonstrated a decrease in TGs to 426 mg/dL after 3 days of an insulin infusion at 0.07 units/kg/hour [20]. A longer treatment duration of 12 days was necessary in a report by Inayat et al., where insulin monotherapy was administered at a rate of 0.1 units/kg/hour in a patient with prediabetes, resulting in a decrease in TGs from 5,047 mg/dL to under 500 mg/dL [10]. None of these studies discussed the use of heparin, and it is unclear whether subcutaneous heparin was administered in any of the above cases. In three reports where heparin use was described in patients without diabetes, total daily doses were similar to typical doses used for venous thromboembolism prophylaxis [13, 17, 22] and less than or equal to the dose used in our patient case. Interestingly, one reported monotherapy with heparin at 15,000 units/day with a resolution of hypertriglyceridemia (8,500 mg/dL to 408 mg/dL) in 3 days; [22] see (Table 1).

Unlike other studies in patients without diabetes that reported a rapid and substantial correction of TG levels within the first few hours/days of therapy [9, 10, 13, 15, 20, 22], we observed a gradual decrease in TG levels. We did not observe the same magnitude of TG lowering (upwards of 90% reduction in most published cases) despite the use of a higher insulin infusion rate. A maximum reduction of approximately 82% from baseline was observed after 7 days of continuous IV insulin at rates up to 19 units/hr. Notably, a 72% and 80% reduction were also observed on hospital days 4 and 5 at an infusion rate of 15 units/hr, suggestive of a plateau effect. This may be indicative of the need for longer durations of insulin infusions at moderate rates as opposed to aggressive titrations. The observation of a similar TG level reduction from the baseline with less aggressive insulin infusion rates (15 units/hr vs 25 units/hr) may suggest that lower infusion rates of insulin are as efficacious as higher rates in the management of hypertriglyceridemia with a reduced risk for hypoglycemia. In addition to the risk of hypoglycemia, there is a risk of inducing hypokalemia with prolonged/aggressive insulin therapy. In our case, the patient still experienced hypokalemia despite daily potassium supplementation.

Another area of uncertainty was the significance of heparin therapy in the reduction of TG levels. From our study, we observed an increase in the serum TG when insulin therapy was discontinued, even though heparin therapy was continued for two days, but it is unclear if the increase observed was attenuated by the use of heparin. Given no reports of increased bleed risk with heparin in this patient population and the often-concurrent indication for thromboprophylaxis, it seems prudent to use subcutaneous heparin at doses to prevent thromboembolism for the potential added benefits in patients with HTGIP.

As the patient received combination therapy, the efficacy of each individual medication is unknown. The patient's lack of adherence to oral TG-lowering agents prior to admission adds even more difficulty in separating the effects of the various treatment components. Though it is reasonable to predict that the reinitiation of omega-3 fatty acid and statin therapy did not contribute significantly to the acute decline in TG levels, we are unable to determine the degree to which they may have assisted with the resolution of hypertriglyceridemia over the hospital course. There is no defined duration of treatment for managing hypertriglyceridemia. Most reports show a fast resolution of symptoms (usually within hours to days), but our patient required more aggressive therapy and we were unable to attain the initial TG goal of <500 mg/dL. While the patient's serum TGs remained around 1,000 mg/dL upon discharge, the “resistance” to conventional treatments was likely multifactorial with a contribution from her history of familial hypertriglyceridemia, obesity, prediabetes, and medication nonadherence prior to admission. The patient reported improved adherence to her medications during a 1-month postdischarge follow-up encounter where her triglycerides were 495 mg/dL.

In this case report, we demonstrate the successful treatment of acute pancreatitis secondary to hypertriglyceridemia in a patient without diabetes with intravenous insulin infusions and subcutaneous heparin, providing additional support for the use of this management strategy among patients without diabetes. The medical management of hypertriglyceridemia-induced acute pancreatitis is more robust in patients with diabetes, but in general, approaches to therapy vary in the literature and are further complicated by the lack of standardization of treatment. Furthermore, research is needed to develop treatment recommendations that differentiate between patients with and without diabetes, especially with regard to the appropriateness and dosing, as well as the duration of insulin, dextrose, potassium, heparin, and combination therapies.

## Figures and Tables

**Table 1:** Studies evaluating insulin and/or heparin for hypertriglyceridemia management in adult patients.

Study	Number of patients (without diabetes)	Age (years)	Gender	Initial TG (mg/dL)	Treatment	Posttreatment TG (mg/dL)	Duration of treatment (days)	Hypo-kalemia	Hypo-glycemia
Monotherapy insulin									
Coskun et al. [8]^*∗*^	12 (4)	46	8 male 4 female	Mean 1,140	Insulin infusion unspecified rate	Mean 492	Mean 3	No	No
Mikhail et al. [18]	1	38	1 female	10,560	Subcutaneous insulin sliding scale every 4 hours	712	3	No	No
Khan et al. [15]	1 (1)	44	1 female	3,525	Insulin infusion unspecified rate	973	1	No	No
Gayam et al. 2018 [9]	1 (1)	48	1 male	>10,000	Insulin infusion 1–2 units/kg/day	<300	8	No	No
Reed et al. [20]	1 (1)	34	1 female	3,496	Insulin infusion 0.07 units/kg/hour	427	3	No	Yes
Inayat et al. [10]	1 (1)	39	1 male	5,047	Insulin infusion 0.1 units/kg/hour	<500	12	No	No

Monotherapy heparin									
Sleth et al. [22]	1 (1)	28	1 female	8,500	Intravenous heparin 15,000 units/day then subcutaneous tinzaparin 2,500 units/day	408	3 days of heparin	No	No

Combination therapy									
Jain and Zimmerschied [12]	1	54	1 male	10,320	Insulin infusion and intravenous heparin unspecified rates	<2,500	3	No	No
Jain et al. [13]	1 (1)	46	1 male	18,220	Insulin infusion unspecified rate and subcutaneous heparin 10,000 units/day	470	4	No	No
Kuchay et al. [17]^§^	4 (1)	36	1 female	1820	Insulin infusion 2–12 units/hour and subcutaneous heparin 60 units/kg every 8 hours	534	3	No	No
Jin et al. [14]^*∗*^^§^	34	34.6	18 male 16 female	Mean 3,089	Insulin infusion 0.1 units/kg/hour and intravenous heparin 10–15 units/kg/hour	Mean 772	Mean 5	Yes	No
Camargo-Mendoza and Bustos-Calvo [7]	1	30	1 male	6,700	Insulin infusion and heparin 80 unit/kg bolus followed by infusion unspecified rates	454	4	No	No
Patel [19]	1	42	1 female	>5,000	Insulin infusion 0.1 units/kg/hour and intravenous heparin 600 units/hour	423	Unspecified	No	No

^*∗*^Data from patients without diabetes are not decipherable from the study. ^§^No bleeding events were reported in the study.

## Data Availability

The data used to support the findings of this study are included in the tables and are also available from the corresponding author upon request.
